# Therapeutic novelties in migraine: new drugs, new hope?

**DOI:** 10.1186/s10194-019-0974-3

**Published:** 2019-04-17

**Authors:** Thien Phu Do, Song Guo, Messoud Ashina

**Affiliations:** Danish Headache Center and Department of Neurology, Rigshospitalet Glostrup, Faculty of Health Sciences, University of Copenhagen, Copenhagen, Denmark

**Keywords:** Migraine, Randomized clinical trial, Efficacy, Tolerability, Adverse event, Ditan, Gepant, Antibody

## Abstract

**Background:**

In the past decade, migraine research has identified novel drug targets. In this review, we discuss recent data on emerging anti-migraine therapies.

**Main body:**

The development of ditans, gepants and anti-calcitonin gene-related peptide monoclonal antibodies for the treatment of migraine is one of the greatest advances in the migraine field. Lasmiditan, rimegepant and ubrogepant will extend our therapeutic armamentarium for managing acute migraine attacks when triptans are not effective or contraindicated due to cardiovascular disorders. The monoclonal antibodies are migraine specific prophylactic drugs with high responder rates and favorable adverse event profiles. Furthermore, they offer convenient treatment regimens of 4- or 12-week intervals.

**Conclusion:**

Collectively, novel migraine therapies represent a major progress in migraine treatment and will undoubtedly transform headache medicine.

## Introduction

The last ten years have witnessed remarkable discoveries in migraine research [1, 2] and migraine therapy is currently undergoing tremendous development. Based on these discoveries, novel targeted acute and preventive therapies have emerged including ditans (5-HT_1F_ receptor agonists), gepants (calcitonin gene-related peptide (CGRP) receptor antagonists) and anti-CGRP monoclonal antibodies (mAbs). Novel therapies signify a paradigm shift in the migraine management and bring new hope to patients and clinicians. This review provides an overview of new drugs for both acute and prophylactic treatments of migraine, covering studies on clinical evidence, tolerability, and the different stages of clinical development.

### Novel acute treatment targets

#### 5-HT_1F_ receptor agonists (ditans)

Triptans are 5-HT_1B_/_1D_ receptor agonists with some affinity for the 5-HT_1F_ receptor subtype and commonly used as acute anti-migraine drugs [3]. The rationale for the development of triptans was based on the vasoconstricting effect via the 5-HT_1B_ receptor subtype [4]. However, some studies have questioned the role of vasoconstriction in anti-migraine effect of triptans [5]. Therefore, drug pharmacological studies have focused on the 5-HT_1D_ [6, 7] and 5-HT_1F_ receptors that do not a vasoconstrictive effect [8–10]. These receptors are interesting drug targets as triptans are contraindicated in migraine patients with coexisting cardiovascular disorders [11–15]. The 5-HT_1D_ subtype is expressed in the human trigeminal ganglion and co-localize with CGRP [6]. A phase II trial investigated the efficacy of 5-HT_1D_ agonists, but did not meet its primary endpoints and its development was discontinued [7]. Therefore, drug discovery programs shifted focus to the 5-HT_1F_ subtype. This receptor subtype is located in the trigeminal ganglion, the trigeminal nucleus caudalis and cephalic blood vessels, but importantly, activation of this receptor do not constrict blood vessels [8–10]. Interestingly, sumatriptan and naratriptan binds to the 5-HT_1F_ receptor with a high affinity [9]. Based on these studies, 5-HT_1F_ agonists have been developed and categorized as a new drug class: ditans. Studies of ditans in preclinical models suggested an involvement in the modulation of dural neurogenic inflammation and the trigeminovascular system, establishing the 5-HT_1F_ receptor as a potential target for migraine treatment [16]. Three compounds exist, LY 344864, LY334370 and lasmiditan, but only the last two have been tested in humans. While LY334370 demonstrated a clinical effect in a proof of concept study [17], the development of LY334370 was terminated due to hepatic toxicity in animal models [18]. Accordingly, only lasmiditan is still undergoing clinical trials (Table 1).

**Table 1:** Overview of ditans in alphabetical order

Drug	Status
Alniditan	Development terminated
Lasmiditan (COL-144)	Phase III clinical trials
LY-334370	Development terminated

**Fig. 1:**
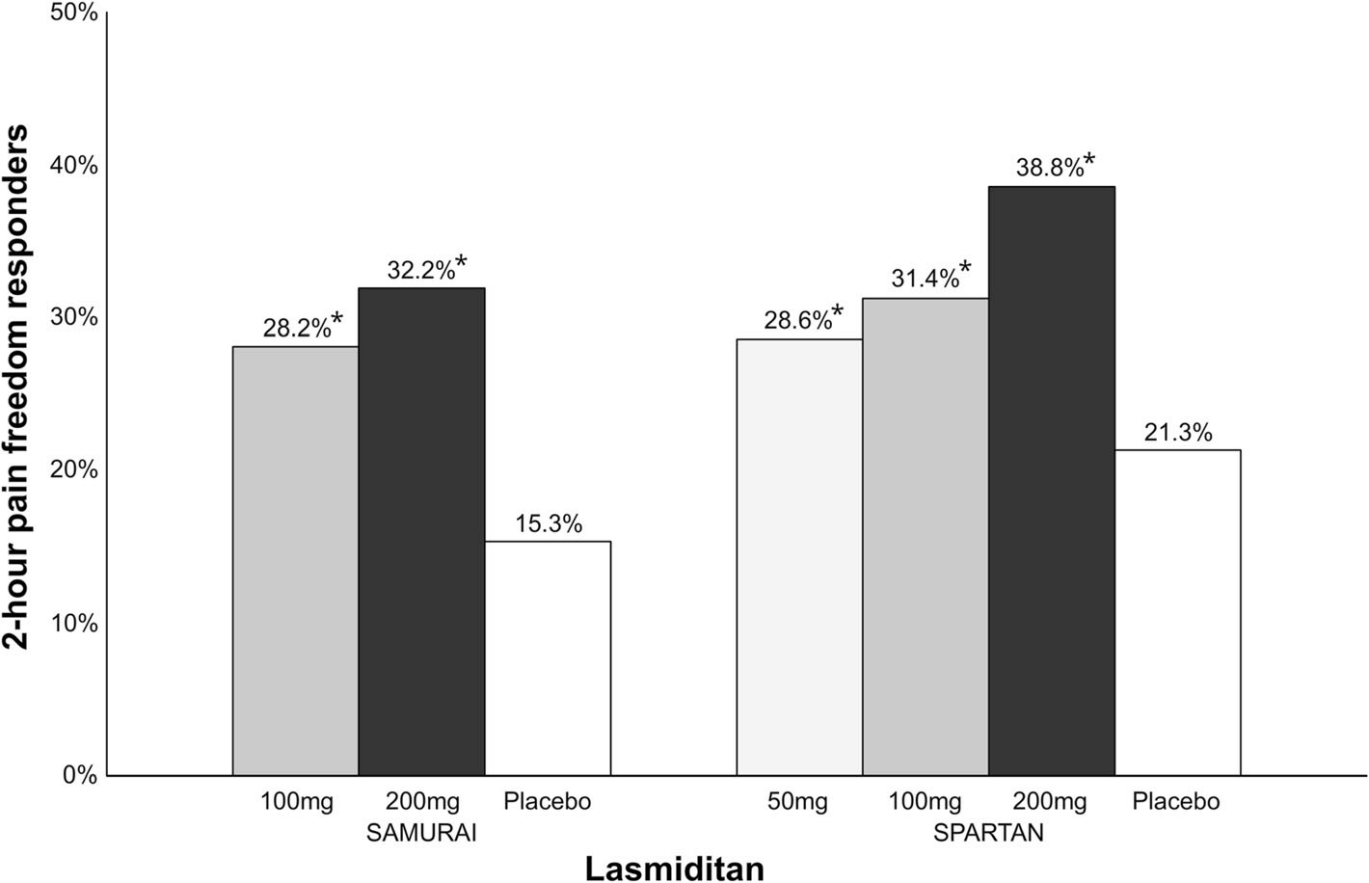
Overview of patients (%) achieving 2-h pain freedom in lasmiditan phase III clinical trials with different doses. A darker bar indicates a higher dose. *vs. placebo, *p* < 0.001

Lasmiditan is a 5-HT_1F_ receptor agonist [19] which is administrated orally in 50–200 mg doses and it has a t_max_ of 1.5–2.5 h [20]. Of the three phase III clinical trials (SAMURAI, NCT02439320 [21]; SPARTAN, NCT02605174 [22]; GLADIATOR, NCT02565186 [23]), to date only one (SAMURAI [24]) has been published [24]. Preliminary data from press releases for the remaining two are presented as following [25, 26] (Fig. 1).

In SAMURAI, 2231 patients were randomized to oral intake of lasmiditan 100 mg, 200 mg or placebo [24]. This trial excluded patients with known coronary artery disease, clinically significant arrhythmia or uncontrolled hypertension. The percentage of patients with 2-h pain freedom was 28.2% (vs. placebo, *p* < 0.001) in the 100 mg group, 32.2% (vs. placebo, *p* < 0.001) in the 200 mg group and 15.3% in the placebo group [27]. The percentage of patients with freedom from most bothersome symptom at 2-h was 40.9% (vs. placebo, *p* < 0.001) in the 100 mg group, 40.7% (vs. placebo, *p* < 0.001) in the 200 mg group compared to 29.5% in the placebo group. The most common adverse events were dizziness and paresthesia and both mild to moderate intensity. Dizziness occurred in 11.9% of the 100 mg group and 15.4% of the 200 mg group. Paresthesia occurred in 5.7% of the 100 mg group and 7.6% of the 200 mg group compared to 3.1% and 2.1% in the placebo group. No serious adverse events occurred.

In SPARTAN, patients were randomized to 50 mg, 100 mg, 200 mg or placebo (number of patients included not reported) [25]. This trial did not exclude patients with known coronary artery disease, clinically significant arrhythmia or uncontrolled hypertension. The percentage of patients with 2-h pain freedom was 28.6% (vs. placebo, *p* = 0.003) in the 50 mg group, 31.4% (vs. placebo, *p* < 0.001) in the 100 mg group, 38.8% (vs. placebo, *p* < 0.001) in the 200 mg group and 21.3% in placebo group. Percentage of patients with freedom from most bothersome symptom at 2-h was 40.8% (vs. placebo, *p* = 0.009) in the 50 mg group, 44.2% (vs. placebo, *p* < 0.001) in the 100 mg group, 48.7% (vs. placebo, *p* < 0.001) in the 200 mg group and 33.5% in the placebo group. Adverse events included dizziness, paresthesia, somnolence, fatigue, nausea and lethargy.

The open-label trial GLADIATOR included patients from the prior SAMURAI and SPARTAN trials [26]. Patients were randomized to receive 100 mg or 200 mg to treat up to eight attacks per month (number of patients included not reported, expected a total of 2580). The primary goal was to evaluate the proportion of patients and attacks associated with any adverse events and specific adverse events. Adverse events occurred in 19% in the 100 mg group and 20% in the 200 mg group. The most common adverse events included dizziness and paresthesia.

**Fig. 2:**
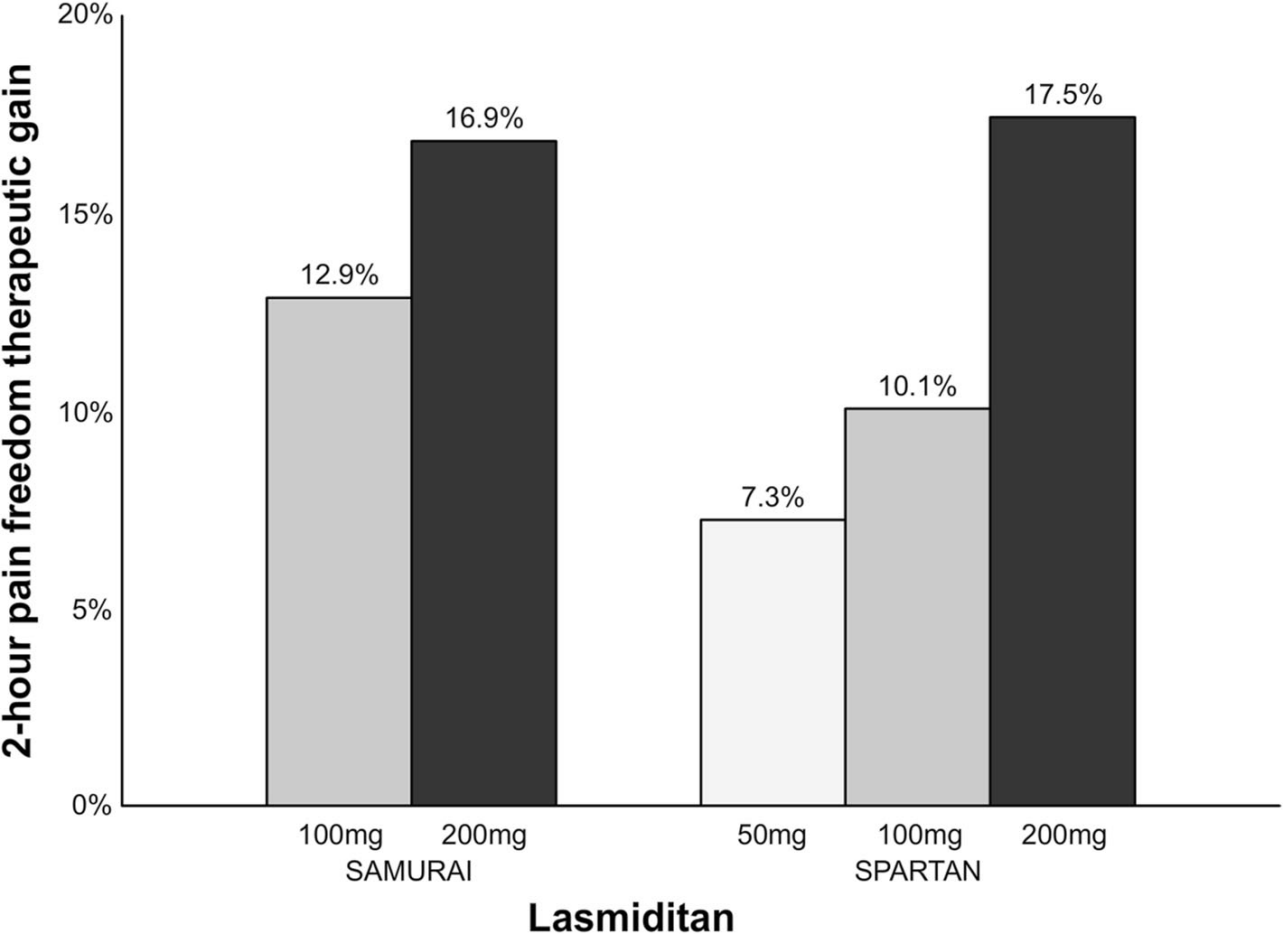
Overview of the therapeutic gain* in 2-h pain freedom with lasmiditan. A darker bar indicates a higher dose. *Therapeutic gain is defined as the difference between percentage of responders in active group compared to percentage of responders in placebo group

Collectively, randomized controlled trials (RCTs) support the use of lasmiditan for the acute treatment of migraine. The percentage of patients with 2-h pain freedom in trials ranges from 28.2–38.8% (Fig. 1). Furthermore, the therapeutic gain (the placebo-subtracted response) for lasmiditan 200 mg is 16.9–17.5% which seems similar to sumatriptan of 16–21% for doses 50–100 mg (Fig. 2) [28]. Approximately 20% of patients report adverse events most commonly dizziness and paresthesia after intake of lasmiditan 100–200 mg [26]. Overall, the results of these trials demonstrate that lasmiditan is efficacious and well tolerated in patients with a high level of cardiovascular risk factors. In the future, lasmiditan will likely be approved as second-line treatment if patients failed with triptans or first line anti-migraine treatment in patients with cardiovascular risk (or documented cardiovascular disease). Lasmiditan is expected to be approved by the U.S. Food and Drugs Administration (FDA) in 2019.

#### CGRP receptor antagonists (gepants)

**Fig. 3:**
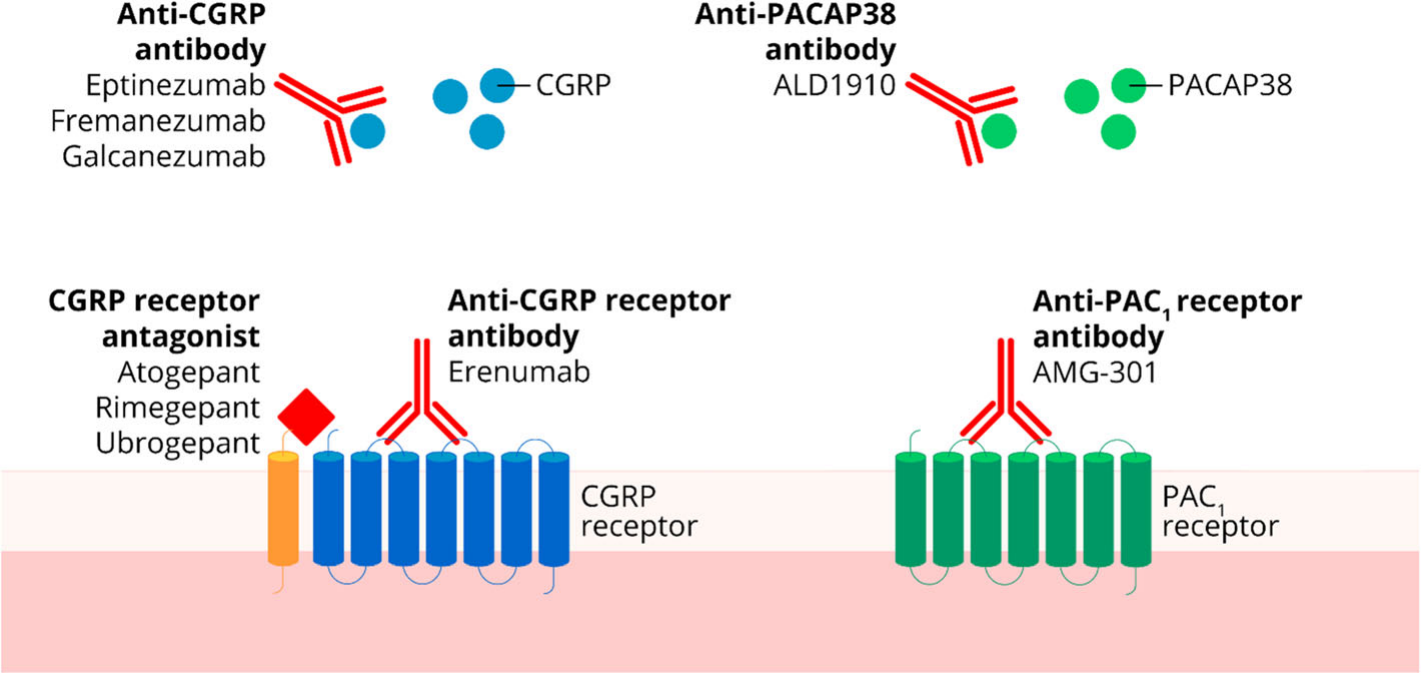
Overview of the therapeutic novelties targeting the calcitonin gene-related peptide (CGRP) and pituitary adenylate cyclase-activating polypeptide/pituitary adenylate cyclase 1 (PACAP/PAC_1_) pathways developed for migraine

Small molecule CGRP receptor antagonists are a novel drug class called gepants (Table 2, Fig. 3). In 2004, the first proof of concept study reported that olcegepant had a clinical effect in humans, but this compound was never commercialized as it cannot be orally administrated [29]. Seven gepants have been developed for the treatment of migraine, but some of the drug development programs have since been terminated [30]. While telcagepant demonstrated a clinical effect, development ceased due to a hepatotoxicity risk [30]. This is believed to be due to a liver toxic metabolite that is not formed by other gepants [31]. Currently, two gepants are in phase III clinical trials for the acute treatment of migraine: rimegepant and ubrogepant (Table 2).

**Table 2:** Overview of gepants for the treatment of migraine in alphabetical order

Drug	Status
Atogepant (AGN-241689, MK-8031)	Phase III clinical trials (prophylactic treatment)
BI 44370	Development terminated
MK-3207	Development terminated
Olcegepant (BIBN4096BS)	Development terminated
Rimegepant (BMS-927711, BHV3000)	Phase III clinical trials (acute treatment); phase II clinical trials (prophylactic treatment)
Telcagepant (MK-0974)	Development terminated
Ubrogepant (MK-1602)	Phase III clinical trials (acute treatment)

**Fig. 4:**
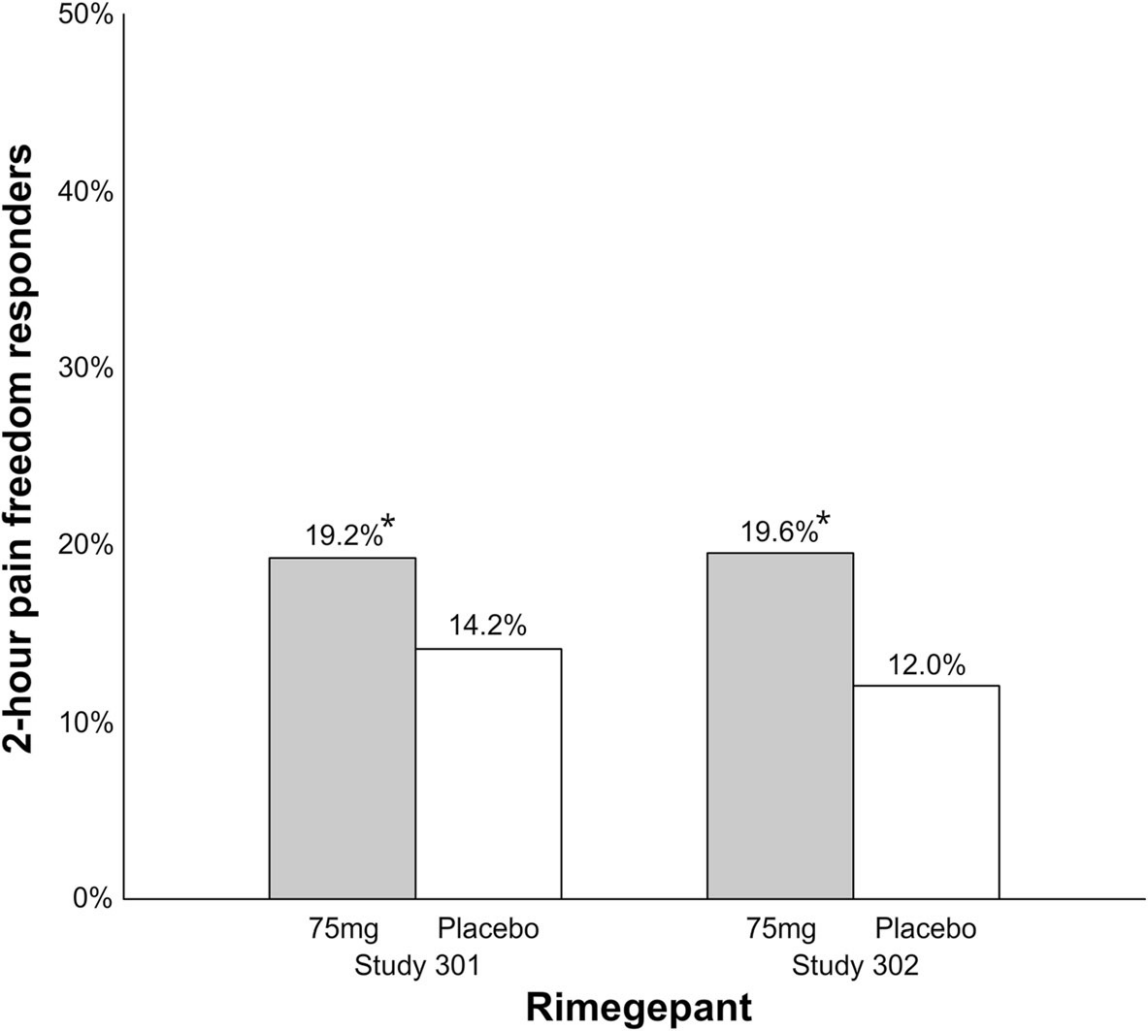
Overview of patients (%) achieving 2-h pain freedom in rimegepant phase III clinical trials. *Study 301; vs. placebo, *p* < 0.003. Study 302; vs. placebo, *p* < 0.001

Rimegepant is a CGRP receptor antagonist [32] that is administrated orally with a 75 mg dose and it has a t_max_ of two hours [33]. Preliminary data from two phase III clinical trials have been reported in press releases but not yet published in peer-reviewed journals [34, 35]. Rimegepant was administrated as a 75 mg oral dose in the two trials (Fig. 4). Preliminary results show that 19.2% (vs. placebo, *p* < 0.003) of 543 patients and 19.6% (vs. placebo, *p* < 0.001) of 537 patients achieve 2-h pain freedom compared to 14.2% of 541 patients and 12% of 535 patients with placebo, respectively [34]. Interestingly, the percentage of patients achieving pain freedom is reported to increase over time with 66% achieving pain freedom at 8 h compared to 47% in placebo group [35]. Freedom from most bothersome symptom was 36.6% (vs. placebo, *p* < 0.002) and 37.6% (vs. placebo, *p* < 0.0001) in the two trials compared to 27.7% and 25.2% with placebo, respectively. Rimegepant had no effect on hepatic function. The number of patients with adverse events has not been reported from either trial but the most common adverse events were nausea (1.4% in active vs. 1.1% in placebo group) and urinary tract infections (1% in active vs. 0.7% in placebo group). Overall adverse event rate is reported to be similar to placebo.

**Fig. 5:**
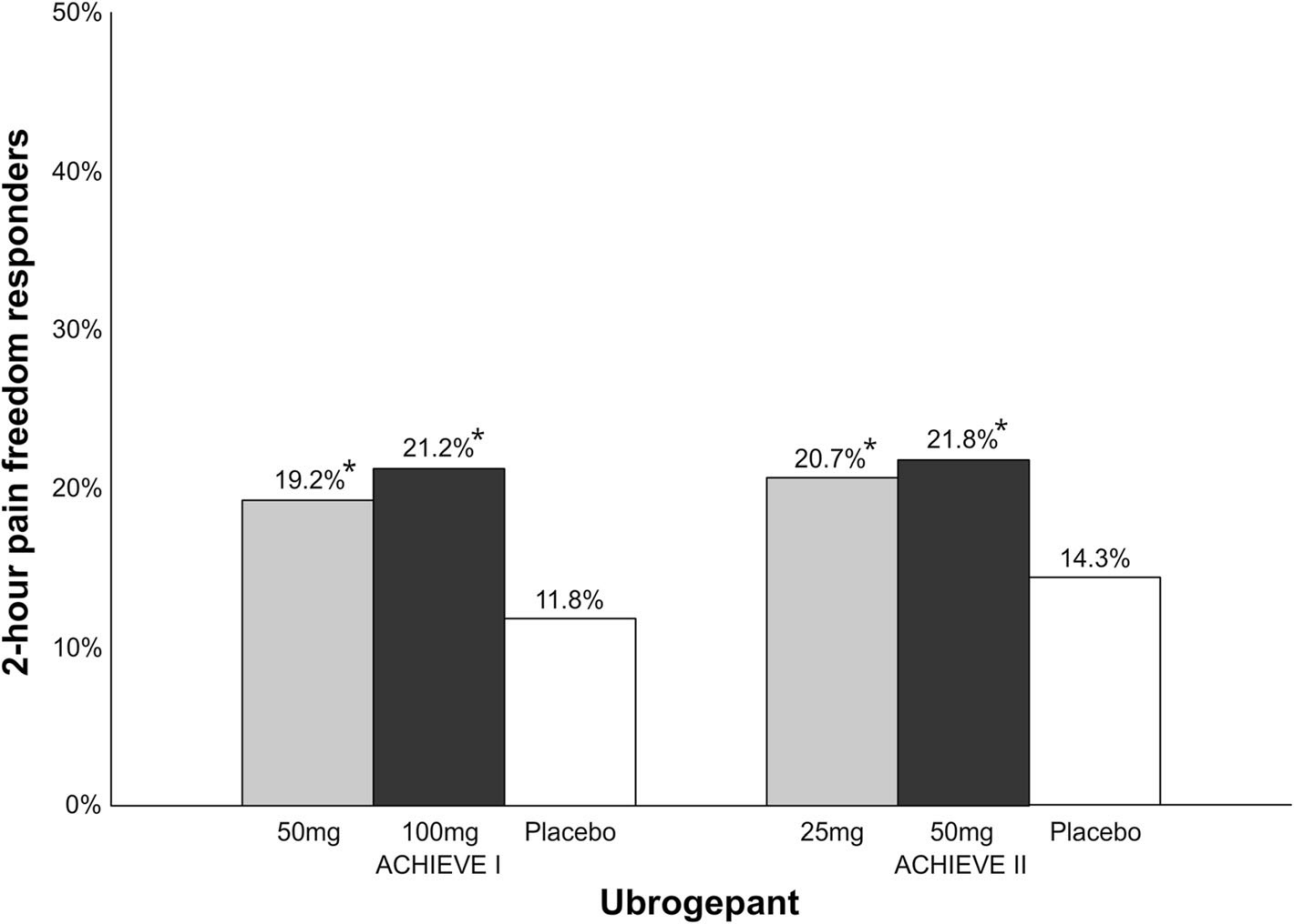
Overview of patients (%) achieving 2-h pain freedom in ubrogepant phase III clinical trials. *ACHIEVE I; 50 mg vs. placebo, *p* = 0.0023; 100 mg vs. placebo, *p* = 0.0003. ACHIEVE II; 25 mg vs. placebo, *p* = 0.0285; 50 mg vs. placebo, *p* = 0.0129

Ubrogepant is a CGRP receptor antagonist that is administrated orally with 25–100 mg doses and it has a t_max_ of 0.7–1.5 h [36]. Preliminary data from two phase III clinical trials (ACHIEVE I and ACHIEVE II) have been reported in press releases but not yet published in peer-reviewed journals [37, 38]. In ACHIEVE I, 1327 patients were randomized 1:1:1 to ubrogepant 50 mg, ubrogepant 100 mg or placebo (Fig. 5) [37]. The percentage of patients with 2-h pain freedom was 19.2% (vs. placebo, *p* = 0.0023) in the 50 mg group, 21.2% (vs. placebo, *p* = 0.0003) in the 100 mg group and 11.8% in the placebo group. Freedom from most bothersome symptom at 2-h was 38.6% (vs. placebo, p = 0.0023) and 37.7% (vs. placebo, p = 0.0023) compared to 27.8% for placebo, respectively. No hepatoxicity was reported after intake of ubrogepant. The most common adverse events were nausea, somnolence, and dry mouth all reported with a frequency lower than 5%. In ACHIEVE II, 1686 patients were randomized 1:1:1 to ubrogepant 25 mg, ubrogepant 50 mg or placebo (Fig. 4) [38]. The percentage of patients with 2-h pain freedom was 20.7% (vs. placebo, *p* = 0.0285) in the 25 mg group, 21.8% (vs. placebo, *p* = 0.0129) in the 50 mg group and 14.3% in the placebo group. Freedom from most bothersome symptom at 2-h was 34.1% (vs. placebo, *p* = 0.0711) and 38.9% (vs. placebo, p = 0.0129), respectively, compared to 27.4% for placebo with the 25 mg dose not being statistically significant compared to placebo. There was no signal of hepatic toxicity in this trial. The most common adverse events were nausea and dizziness all reported with a frequency lower than 2.5%.

**Fig. 6:**
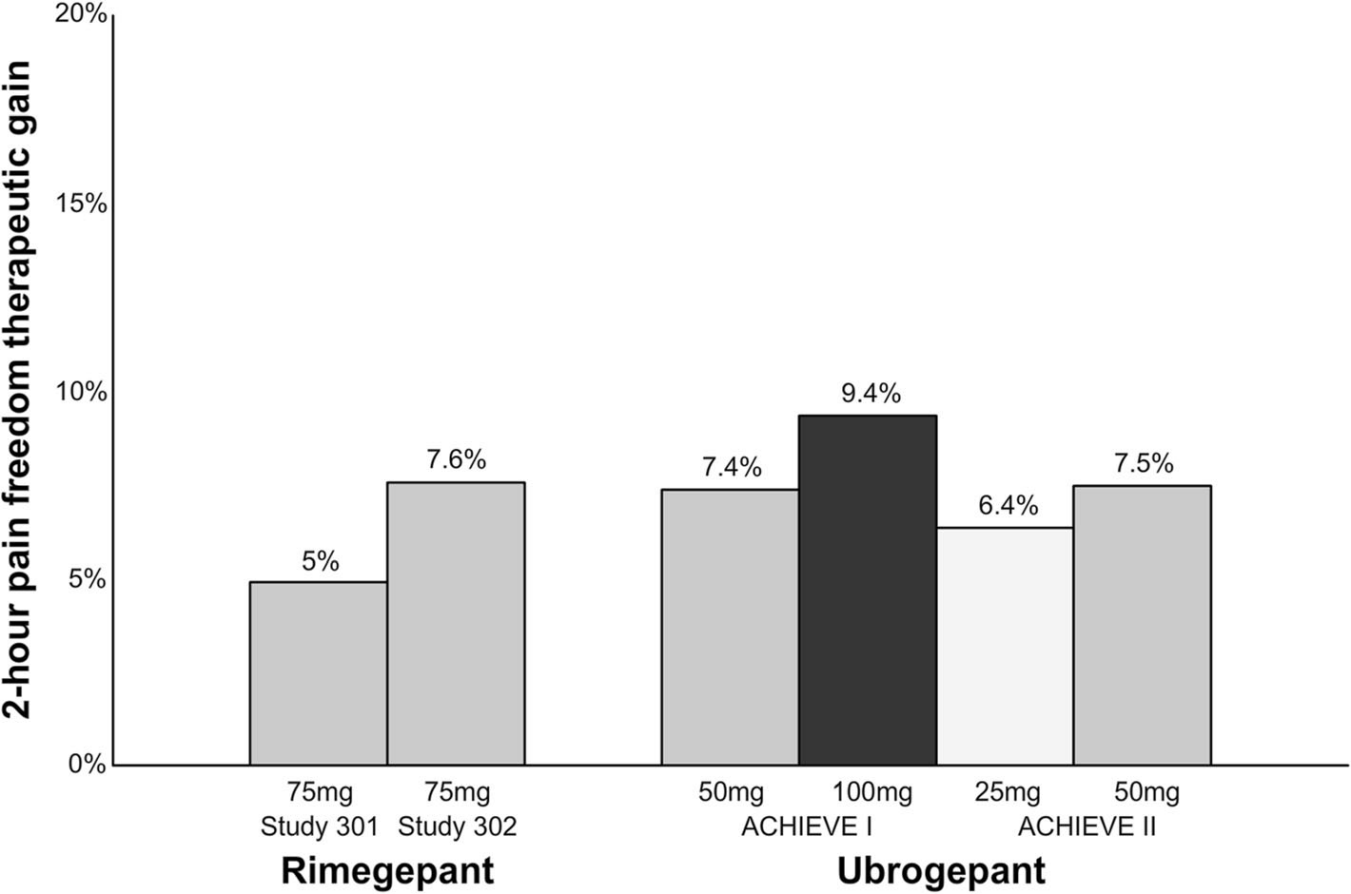
Overview of the therapeutic gain* in 2-h pain freedom with gepants. A darker bar indicates a higher dose. *Therapeutic gain is defined as the difference between percentage of responders in active group compared to percentage of responders in placebo group

Collectively, RCTs demonstrated efficacy of gepants for the acute treatment of migraine. The percentage of patients with 2-h pain freedom ranges from 19.2–19.6% with rimegepant and 19.2–21.8% with ubrogepant. However, the therapeutic gain for gepants (rimegepant: 5%–7.6%; ubrogepant: 6.4%–9.4%) (Fig. 6) seems to be low, especially compared to sumatriptan (16%–21% [28]) and lasmiditan (7.3%–17.5%) (Fig. 2). In addition, it is lower compared to the therapeutic gain of telcagepant (17% in doses 280–300 mg) [39] and it is unlikely due unoptimized dosage or absorption rate [40]. Previous trials of gepants caused concerns regarding the hepatic safety, but single treatments with rimegepant and ubrogepant were not associated with hepatotoxicity. Since gepants do not constrict cranial arteries [41–43], they, like ditans, can be used as first line anti-migraine treatment in patients with cardiovascular risk (or documented cardiovascular disease) or as second-line treatment if patients failed with triptans. The first gepants are expected to be approved by the FDA in 2019/2020 [44, 45].

### Novel prophylactic treatment targets

#### Gepants

Rimegepant (NCT03732638; phase II/III) and atogepant (NCT02848326, NCT03700320; phase II/III, phase III) are currently undergoing clinical trials in humans for prophylactic treatment of migraine but only data on atogepant has been released [46].

Preliminary data from the phase II clinical trial on atogepant have been reported in press releases [46]. The trial included 834 patients and was designed as a placebo-controlled dose ranging study with doses ranging from atogepant 10 mg once a day to 60 mg twice a day. All doses showed a significant reduction in mean monthly migraine days compared to placebo. The trial raised no concerns regarding hepatic or cardiovascular safety. Efficacy and safety data on atogepant need to be confirmed in phase III clinical trials.

#### Anti-CGRP mAbs

To date, four mAbs targeting the CGRP pathway have been developed (Table 3) and three of them have already been approved for the preventive treatment of migraine [47–49]. In the following we review data derived from recent phase III trials.

**Table 3:** Overview of anti-calcitonin-gene related (CGRP) (receptor) peptide monoclonal antibodies in order by target and alphabetical

Drug	Target	Administration	Interval between administrations	Status
Erenumab (AMG-334)	Receptor	Subcutaneous injection	4 weeks	FDA approved; phase III clinical trials
Eptinezumab (ALD403)	Ligand	Intravenous infusion	12 weeks	Phase III clinical trials
Fremanezumab (TEV-48125)	Ligand	Subcutaneous injection	4 or 12 weeks	FDA approved; phase III clinical trials
Galcanezumab (LY2951742)	Ligand	Subcutaneous injection	4 weeks	FDA approved; phase III clinical trials

*FDA: The US Food and Drug Administration

**Fig. 7:**
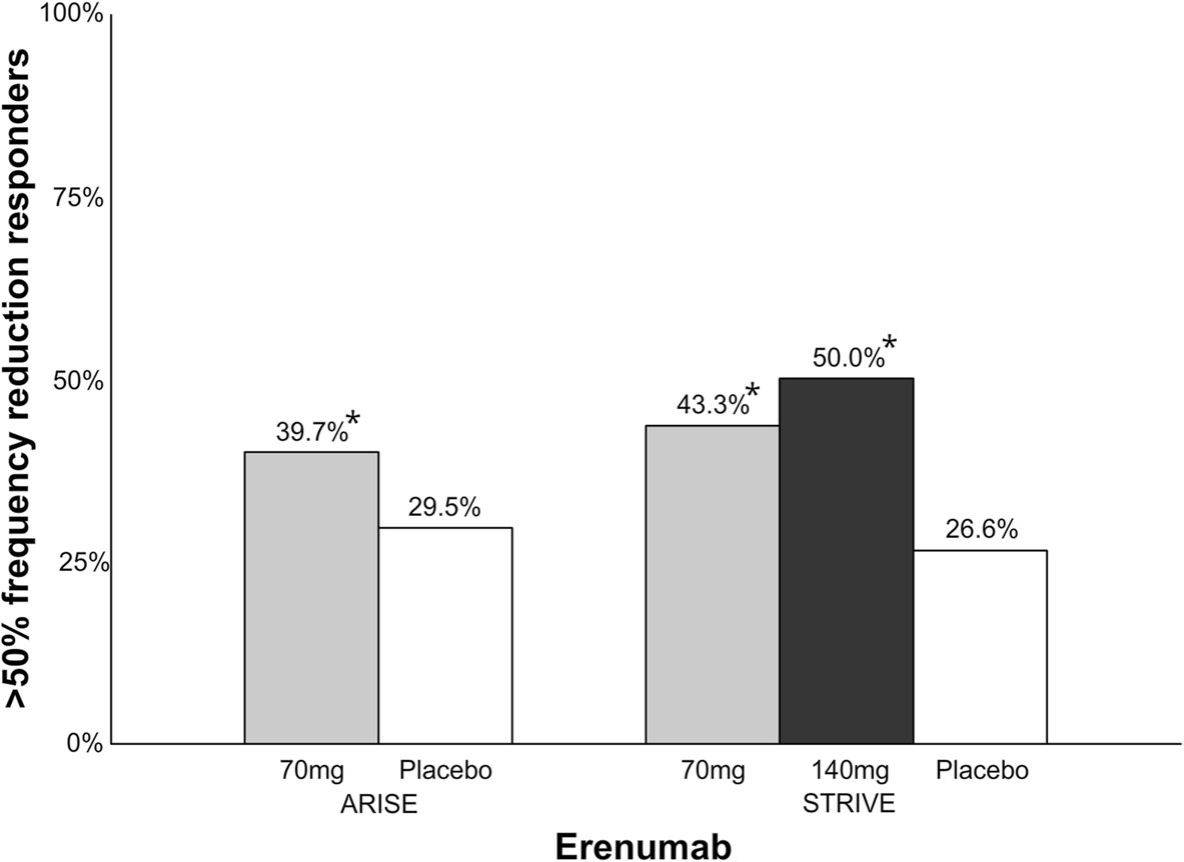
Overview of patients (%) achieving > 50% reduction in migraine days in phase III clinical trials with erenumab. A darker bar indicates a higher dose. *ARISE; 70 mg vs. placebo, *p* = 0.010. STRIVE; 70 mg vs. placebo, *p* < 0.001; 140 mg vs. placebo, *p* < 0.001

Erenumab is a humanized IgG_2_ mAb that targets the CGRP receptor [50, 51] and administered as monthly subcutaneous injections of either 70 mg or 140 mg. The mean t_max_ is 5.5 days and the plasma half-time is approximately 21–23 days [52]. The T_max_ corresponds with an early onset of effect with separation from placebo within the first week of treatment [53]. It has recently been approved for therapeutic use for the preventive treatment of migraine [47]. Data from two phase III clinical trials (ARISE and STRIVE) are presented in the following (Fig. 7). In ARISE, 577 patients were randomized to monthly injections of 70 mg erenumab or placebo [54]. The percentage of patients achieving a > 50% reduction in monthly migraine days was 39.7% (*p* = 0.010) in the active group and 29.5% in the placebo group. Adverse event rates were similar between erenumab and placebo. In STRIVE, 955 patients were randomized to monthly injections of erenumab 70 mg, 140 mg or placebo [55]. The percentage of patients achieving > 50% reduction in monthly migraine days was 43.3% (vs. placebo, *p* < 0.001) with 70 mg, 50.0% (vs. placebo, p < 0.001) with 140 mg and 26.6% with placebo. There was no difference in adverse events between erenumab and placebo. The trial reported 8.0% of the 70 mg group and 3.2% of the 140 mg group creating anti-erenumab binding antibodies, however, only 5.6% of the patients were available for analysis.

**Fig. 8:**
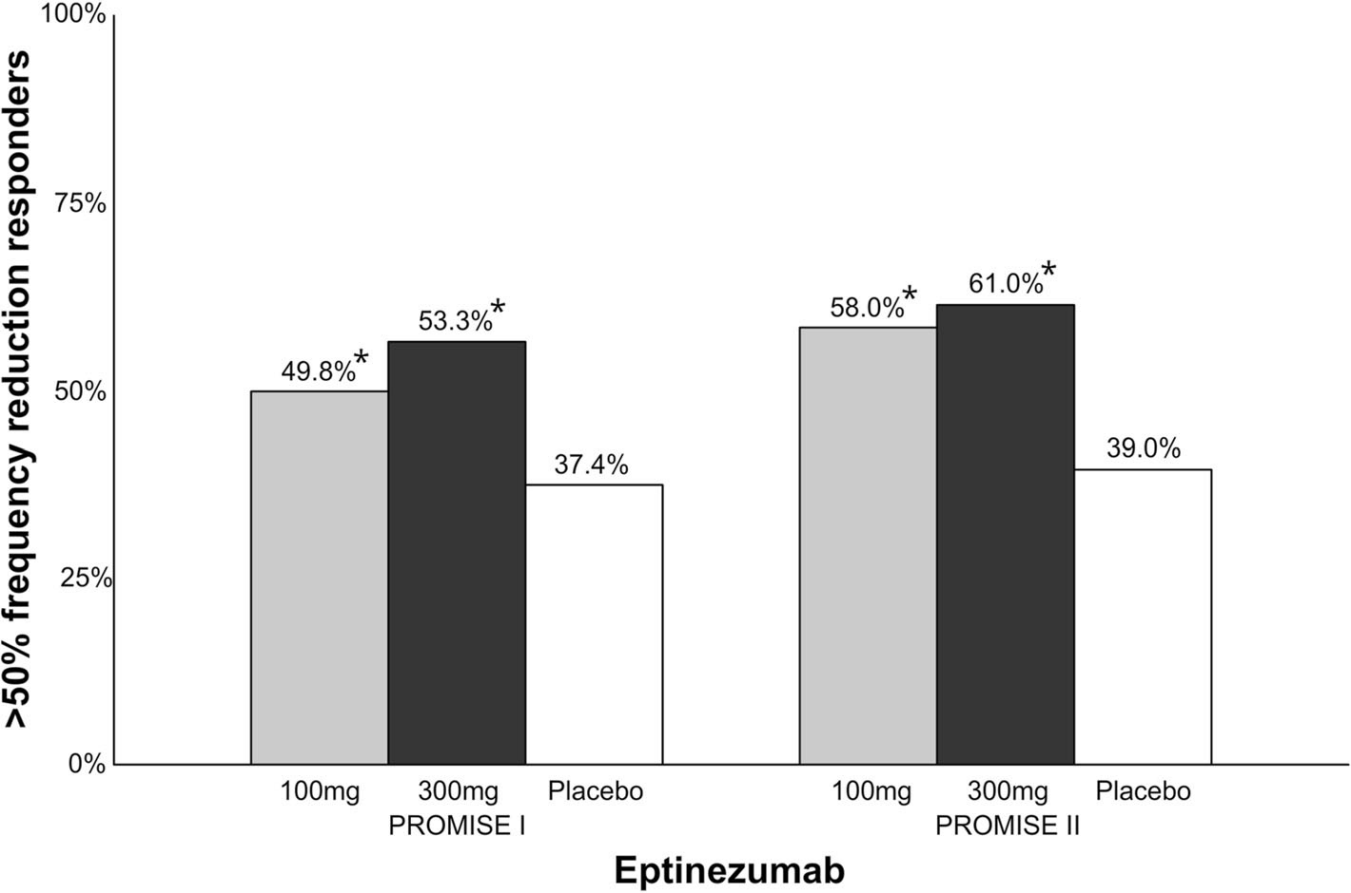
Overview of patients (%) achieving > 50% reduction in migraine days in phase III clinical trials with eptinezumab. A darker bar indicates a higher dose. *PROMISE I; 100 mg vs. placebo, *p* = 0.0085; 300 mg vs. placebo, *p* = 0.0001. PROMISE II; 100 mg vs. placebo, *p* < 0.0001; 300 mg vs. placebo, *p* < 0.0001

Eptinezumab is a humanized IgG_1_ mAb that binds to both α and β forms of the human CGRP ligand [56]. The drug is administrated with intravenous infusions every 12 weeks. The plasma half-time of the drug is 31 days [56]. There is one completed phase III clinical trial (PROMISE I) [57], one ongoing phase III clinical trial (PROMISE II, NCT02974153) [58] and one ongoing safety open-label study (PREVAIL, NCT02985398) [59, 60]. Preliminary data from the PROMISE I [61, 62] and PROMISE II [63] trials (Fig. 8) have been reported in press releases but not yet published in peer-reviewed journals. In PROMISE I, 888 patients were randomized to receive eptinezumab 30 mg, 100 mg, 300 mg or placebo infusions once every 12 weeks [61, 62]. For week 1–12, percentage of patients achieving > 50% reduction in monthly migraine days was 49.8% (vs. placebo, *p* = 0.0085) with 100 mg, 56.3% (vs. placebo, *p* = 0.0001) with 300 mg and 37.4% with placebo [61]. For month 6–12, 70.7% patients had a > 50% reduction in monthly migraine days compared to 58.7% for placebo [62]. Differences between doses were not reported for month 6–12. The most commonly reported adverse events across all eptinezumab groups were upper respiratory infection (10.5%), nasopharyngitis (6.8%), and sinusitis (3.6%). In PROMISE II, 1072 patients were randomized to eptinezumab 100 mg, 300 mg or placebo [63]. The percentage of patients achieving > 50% reduction in monthly migraine days at week 1–12 were 58% (vs. placebo, *p* < 0.0001) with 100 mg, 61% (vs. placebo, p < 0.0001) in 300 mg and 39% with placebo. The incidence of adverse events was not statistically different from the placebo group.

**Fig. 9:**
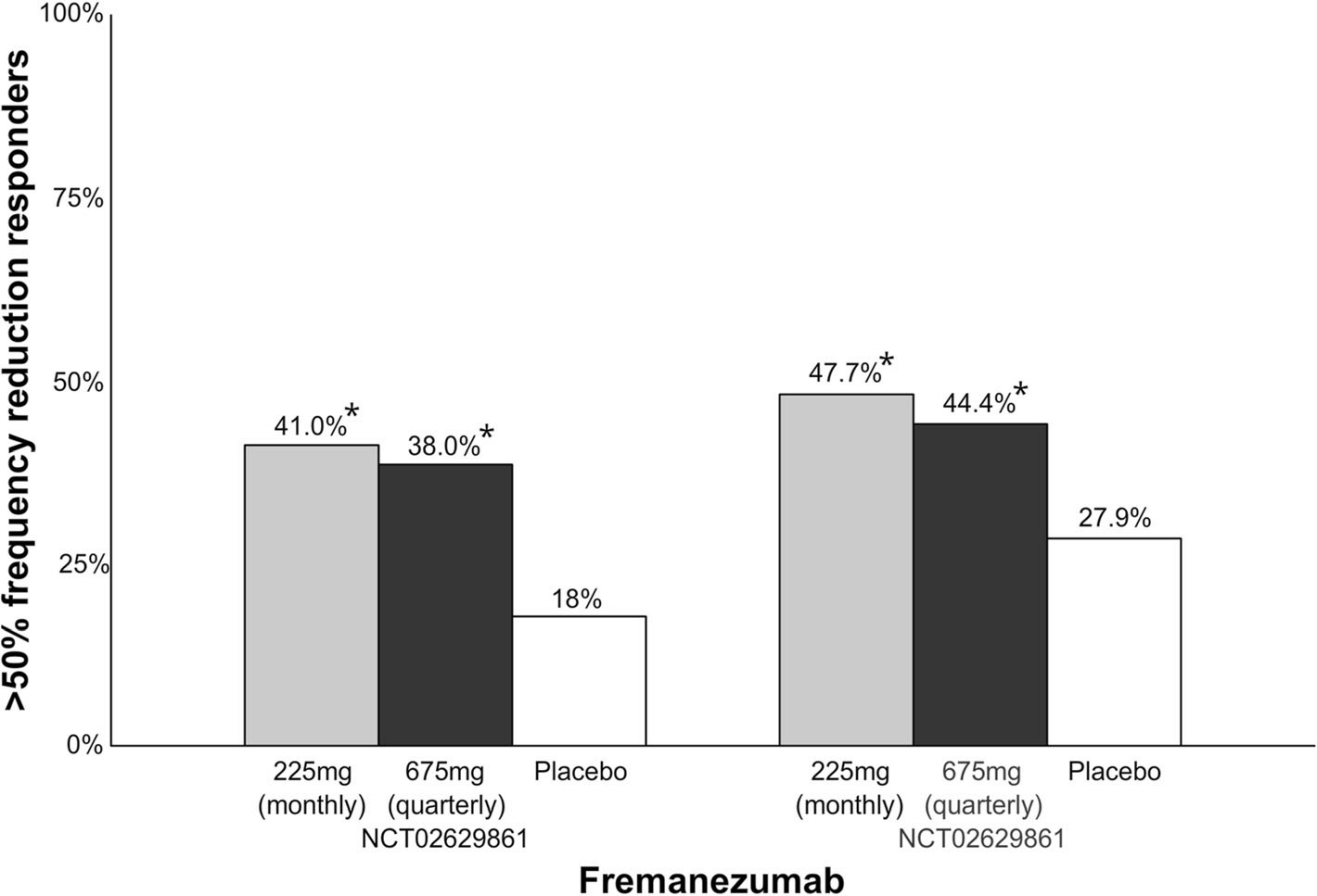
Overview of patients (%) achieving > 50% reduction in migraine days in phase III clinical trials with fremanezumab. A darker bar indicates a higher dose. *NCT02629861 (left); 225 mg vs. placebo, *p* < 0.001; 675 mg vs. placebo, *p* < 0.001. NCT02629861 (right); 225 mg vs. placebo, *p* < 0.001; 675 mg vs. placebo, *p* < 0.001

Fremanezumab is a humanized IgG_2_ mAb that binds to both α and β forms of the human CGRP ligand [64]. Fremanezumab has recently been approved for therapeutic use for the preventive treatment of migraine [48]. The drug is administered as subcutaneous injections with either monthly low-dose 225 mg injections or quarterly high-dose 675 mg injections. The t_max_ is 5–7 days and the plasma half-time of the drug is 31 days. The t_max_ corresponds with an early onset of effect with separation from placebo within the first week of treatment [65]. Results from two phase III clinical trials have been published in peer-reviewed journals (Fig. 9). In a phase III clinical trial [66], 1130 chronic migraine patients were randomized 1:1:1 to monthly low-dose 225 mg injections, quarterly high-dose 675 mg injections or placebo. Percentage of patients with > 50% reduction in monthly migraine days was 41% (vs. placebo, *p* < 0.001) in monthly group, 38% (vs. placebo, p < 0.001) in quarterly group and 18% in the placebo group. Most common adverse event was injection site pain. Two (0.5%) patients in the high-dose quarterly group developed anti-drug antibodies. Similar results were reported in another phase III clinical trial with 875 episodic migraine patients randomized to monthly low-dose 225 mg injections or quarterly high-dose 675 mg injections [67]. Percentage of patients with > 50% migraine frequency reduction was 47.7% (vs. placebo, p < 0.001) in monthly group, in 44.4% (vs. placebo, p < 0.001) quarterly group, and 27.9% in placebo group. Four patients (1.4%) in the low-dose monthly group developed anti-drug antibodies. The most common adverse events were also injection site reactions. In addition, post-hoc analyses show that fremanezumab is safe and effective as add-on treatment for migraine patients who is already on stable doses of other preventive migraine medication [68].

**Fig. 10:**
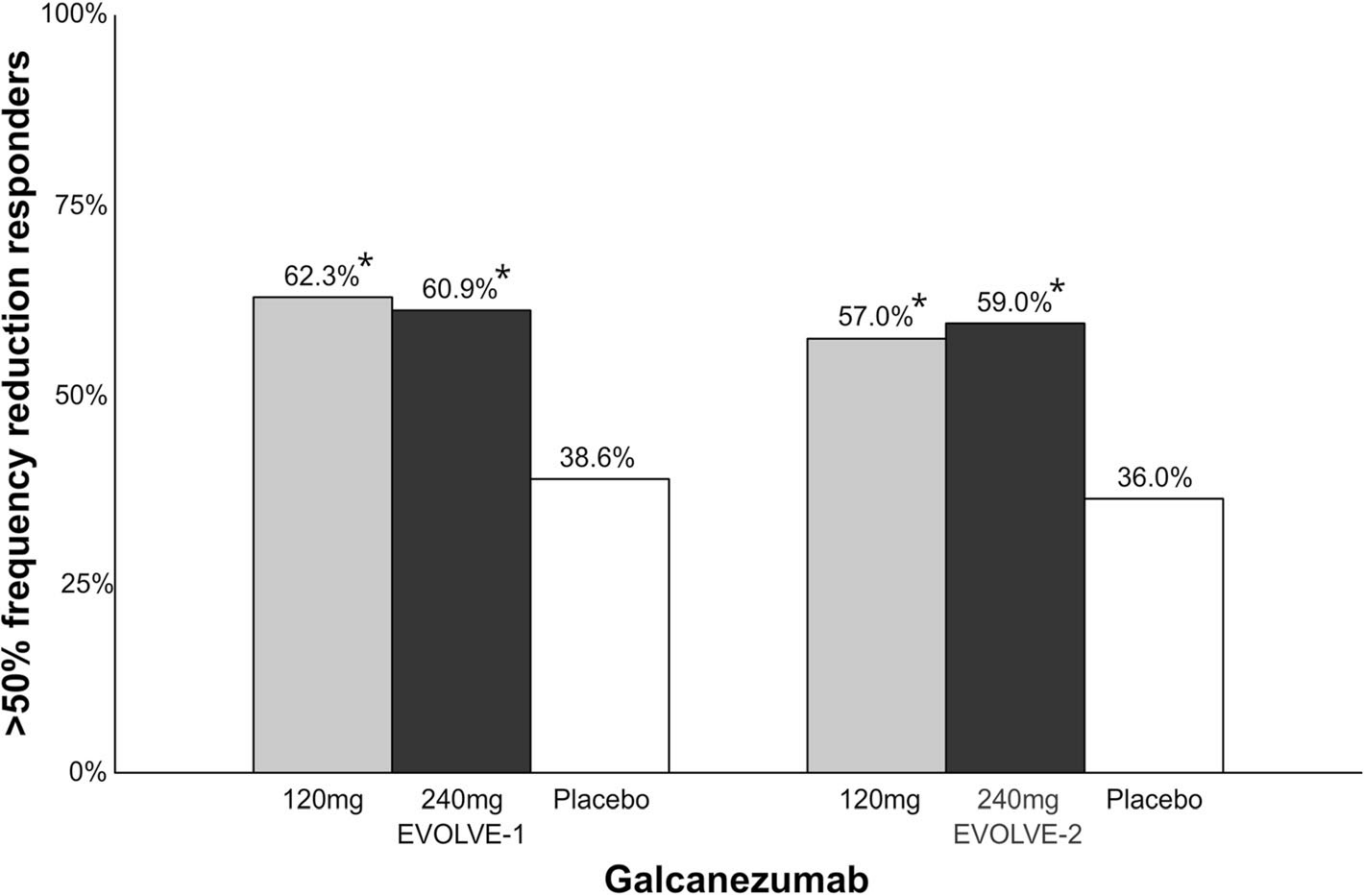
Overview of patients (%) achieving > 50% reduction in migraine days in phase III clinical trials with galcanezumab. A darker bar indicates a higher dose. *EVOLVE-1; 120 mg vs. placebo, *p* < 0.001; 240 mg vs. placebo, *p* < 0.001. EVOLVE-2; 120 mg vs. placebo, *p* < 0.001; 240 mg vs. placebo, *p* < 0.001

Galcanezumab is a humanized IgG_4_ mAb that binds to both α and β forms of the human CGRP ligand. Galcanezumab has recently been approved for the preventive treatment of migraine [49]. The drug is administered as monthly subcutaneous injections. The t_max_ is 7–13 days and the plasma half-time of the drug is 28 days. Results from two phase III clinical trials (EVOLVE-1 and EVOLVE-2) have been published in peer-reviewed journals (Fig. 10). In EVOLVE-1, 1671 patients were randomized 1:1:2 to galcanezumab 120 mg, 240 mg or placebo [69]. Percentage of patients with > 50% migraine frequency reduction was 60.9% (vs. placebo, *p* < 0.001) in 240 mg group, 62.3% (vs. placebo, p < 0.001) in 120 mg group and 38.6% in placebo group. The most common adverse event was injection site reactions. The number of treatment related adverse events was not statistically significant between the active and placebo groups. The percentage of patients who had anti-drug antibodies present after treatment were 5.2% in 240 mg group, 3.5% in 120 mg group and 1.7% in placebo group. In EVOLVE-2, 915 patients were randomized 1:1:2 to galcanezumab 120 mg, 240 mg or placebo [70]. Percentage of patients with > 50% migraine frequency reduction was 59% (vs. placebo, p < 0.001) in 240 mg group, 57% (vs. placebo, p < 0.001) in 120 mg group and 36% in placebo group. The most common adverse event was injection site reactions with a higher rate in the active groups compared to placebo. There was no difference in number of serious adverse events between the groups. The percentage of patients who had anti-drug antibodies present after treatment was 5.1% in 240 mg group, 8.6% in 120 mg group and 0.5% in placebo group. Treatment-emergent anti-drug antibodies had no impact on either safety or efficacy.

**Fig. 11:**
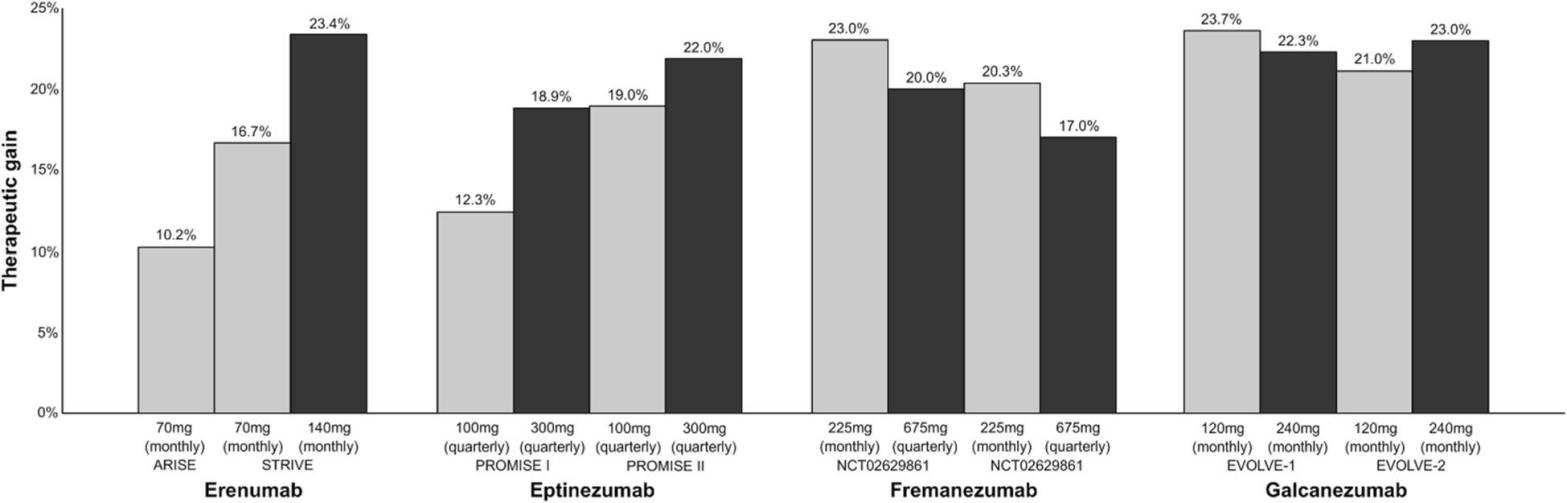
Overview of the therapeutic gain* in percentage of patients with > 50% reduction in migraine days with anti-calcitonin gene-related peptide monoclonal antibodies. A darker bar indicates a higher dose. *Therapeutic gain is defined as the difference between percentage of patients in active group compared to percentage of patients in placebo group

The introduction of anti-CGRP mAbs is a great advancement in migraine treatment because of responder rates with very favorable adverse event profiles. The highest percentage of patients with > 50% reduction in migraine days with each mAb ranges from 47.7%–62%. This suggests a difference in the ratio of responders between the different mAbs. However, the therapeutic gain range is 22–23.7% indicating that anti-CGRP mAbs have a similar efficacy regardless of target (receptor or ligand) and administration form (subcutaneous or intravenous) (Fig. 11). The therapeutic gain with erenumab is increased with higher dosage (Fig. 11). Interestingly, the proportion of patients reaching ≥75% reduction from baseline at 3 months is also statistically significant with anti-CGRP mAbs compared to placebo. This suggests that there are subpopulations who greatly benefit from anti-CGRP mAbs. Efforts should be made to identify these patients and biomarkers that can predict treatment response. The long-term effects (sustained efficacy and safety) of anti-CGRP mAbs remains to be investigated in real world studies. The rate of anti-drug binding antibodies is low in all trials reporting this parameter [55, 66, 67, 69, 70]. Adverse events related to the development of anti-drug antibodies has not been reported in anti-CGRP clinical trials. Whether anti-drug antibodies inactivate the clinical effect depends on the concentration of neutralizing anti-drug antibodies. Consequently, the detection of anti-drug antibodies itself is not a contraindication for treating with anti-CGRP mAbs.

#### Anti-pituitary adenylate cyclase-activating polypeptide mAbs

Pituitary adenylate cyclase-activating polypeptide (PACAP) belongs to the superfamily of glucagon/secretin peptides and two bioactive forms exist, PACAP38 and PACAP27 [71]. PACAP38 exists in the trigeminovascular system and deep brain structures amongst others [72–75]. PACAP38 mediates its effect through three receptors, pituitary adenylate cyclase-activating polypeptide type I (PAC_1_), VPAC_1_ and VPAC_2_. Like the CGRP pathway, these receptors cause an activation of adenylate cyclase leading to increased cAMP production [76]. Vasoactive intestinal peptide (VIP) has a similar structure to PACAP38 and affinity for VPAC_1_ and VPAC_2_ receptors. The two peptides differs in that PACAP38 has a much higher affinity for the PAC_1_ receptor [77]. Furthermore, PACAP38 infusions can cause migraine-like attacks while VIP cannot [78, 79]. Thus, only PACAP38 and the PAC_1_ receptor in this pathway are of interest as drug targets.

There are currently two mAbs, ALD1910 and AMG-301, in development for the PACAP38 pathway (Fig. 3) (Table 4). ALD1910 targets the PACAP38 ligand [80] and AMD-301 targets the PAC_1_ receptor [81]. ALD1910 is undergoing preclinical studies and AMG-301 has recently undergone a phase II clinical trial (NCT03238781). No results of either drug have been released so far but results from the AMG-301 trial is expected to be published mid-2019.

**Table 4:** Overview of anti-pituitary adenylate cyclase-activating polypeptide/pituitaryadenylate cyclase 1 (PACAP/PAC_1_) monoclonal antibodies

Drug	Target	Administration	Interval between administrations	Status
ALD1910	Ligand	N/A	N/A	Preclinical phase
AMG-301	Receptor	Subcutaneous injection	4 weeks	Phase II clinical trials

## Concluding remarks

The development of ditans, gepants and anti-CGRP mAbs for the treatment of migraine is one of the greatest advances in the migraine field. Lasmiditan, rimegepant and ubrogepant will extend our therapeutic armamentarium for managing acute migraine attacks when triptans are not effective or contraindicated due to cardiovascular disorders. The mAbs against CGRP and its receptor have high responder rates with favorable adverse event profiles. Furthermore, the mAbs also offer convenient treatment regimens of 4- or 12-week intervals. These factors will contribute to a better adherence. Given that approximately 40–50% of migraine patients do not respond to mAbs, future studies should focus on identification of biomarkers that can predict treatment response. Collectively, novel migraine therapies represent a major progress in migraine treatment and will undoubtedly transform headache medicine.

## Abbreviations

5-HT receptor: 5-hydroxytryptamine receptor
CGRP: Calcitonin gene-related peptide
mAb: Monoclonal antibody
PAC_1_: Pituitary adenylate cyclase-activating polypeptide type I
PACAP: Pituitary adenylate cyclase-activating polypeptide
RCT: Randomized controlled trial
