# Correction to: Therapeutic novelties in migraine: new drugs, new hope?

**DOI:** 10.1186/s10194-019-1013-0

**Published:** 2019-05-17

**Authors:** Thien Phu Do, Song Guo, Messoud Ashina

**Affiliations:** Danish Headache Center and Department of Neurology, Rigshospitalet Glostrup, Faculty of Health Sciences, University of Copenhagen, Copenhagen, Denmark

**Correction to: J Headache Pain (2019) 20:37**

**https://doi.org/10.1186/s10194-019-0974-3**

After publication of the original article [1], the authors have notified us that the row corresponding to the “Alniditan” drug shouldn’t have been included in Table 1. Table 1 should therefore be presented as below:

**Table 1:** Overview of ditans in alphabetical order

Drug	Status
Lasmiditan (COL-144)	Phase III clinical trials
LY-334370	Development terminated
